# The effect of glucocorticoids on tendon cell viability in human tendon explants

**DOI:** 10.3109/17453670902988386

**Published:** 2009-06-01

**Authors:** Margaret Wan Nar Wong, Wai Ting Lui, Sai Chuen Fu, Kwong Man Lee

**Affiliations:** ^1^Department of Orthopaedics, Traumatology Shatin,ShatinHong Kong; ^2^Lee Hysan Clinical Research Laboratories,, Chinese University of Hong KongShatinHong Kong

## Abstract

**Background and purpose** Previous studies on the culture of human tenocytes have shown that dexamethasone and triamcino-lone reduce cell viability, suppress cell proliferation, and reduce collagen synthesis. However, such cell cultures lack the extracellular matrix and three-dimensional structure of normal tendons, which affects their response to stimuli. We established a human tendon explant culture system and tested the effects of dexamethasone and triamcinolone on cell viability.

**Methods** Primary human tendon explant cultures were prepared from healthy hamstring tendons. Tendon strips were harvested from hamstring tendons and cultured in 24-well plates in Dulbecco’s modification of Eagle’s Medium (DMEM) supplemented with 2% fetal calf serum. The tendon explants were treated with 0 μM (control), 10 μM, or 100 μM dexamethasone sodium phosphate or 0 μM (control), 10 μM, or 100 μM triamcinolone acetonide in DMEM for 96 h. Cell viability was measured by Alamar blue assay before and after glucocorticoid treatment.

**Results** Incubation with 10 μM and 100 μM dexamethasone reduced cell viability in human tendon explants by 35% and 45%, respectively, as compared to a 6% increase in the controls (p = 0.01, mixed-effects ANOVA). Triamcinolone at 10 μM and 100 μM reduced cell viability by 33% and 36%, respectively, as compared to a 9% increase in the controls (p = 0.07, mixed-effects ANOVA).

**Interpretation** Human tendon explant cultures can be used to study the effects of glucocorticoids on human tendon. Dexamethasone and triamcinolone suppress the cell viability of human tendon in its natural 3-dimensional environment with matrix anchorage. Human tendon explant cultures provide a species-specific model for further investigation of the effects of glucocorticoids on the metabolism of the extracellular matrix of human tendon, and on its mechanical properties.

## Introduction

Glucocorticoid injection therapy is commonly used in the treatment of rheumatoid arthritis and soft tissue inflammatory conditions. Glucocorticoids block the production of arachidonic acid, and subsequently prostaglandins, that mediate the inflammatory process. The potent anti-inflammatory action gives good rapid control of symptoms. However, there is concern about the risk of spontaneous tendon rupture after local injection of glucocorticoids. Previous in vitro and in vivo studies performed in animals to evaluate the relationship between glucocorticoid injection and tendon rupture have been inconclusive (Wiggins et al. 1995, Campbell et al. 1996, Tsai et al. 2002). The role of glucocorticoids in tendon rupture remains controversial.

The effects of glucocorticoids are highly cell-, tissue-, and species-specific (Nakano et al. 1993, Bird and Tyler 1994, Yuksel et al. 2001). The effects of glucocorticoids also vary with the growth state of the cell and other interacting factors (Oikarinen et al. 1991). The use of human cells or tissues in the study of the effects of glucocorticoids has a distinct advantage, as it avoids the problem of species specificity. Our previous studies on the effects of glucocorticoids in cultured human tenocytes have shown that dexamethasone and triamcinolone reduce cell viability, suppress cell proliferation, and reduce collagen synthesis (Wong et al. 2003, 2004). We postulated that the suppressed cellular activity and collagen synthesis in human tenocytes may lead to disturbed tendon structure, thus predisposing the tendon to subsequent spontaneous rupture.

Unfortunately, cell cultures lack the extracellular matrix and three-dimensional structure of normal tendon, which affects their response to stimuli (Clark et al. 1997, Fu et al. 2005). The normal heterogeneity of cells is also absent. Tendon explant cultures offer the opportunity to study tendon response under in vitro conditions in which extracellular matrix, cell heterogeneity, and cell anchorage are preserved (Vogel and Hernandez 1992, Yoshikawa and Abrahamsson 2001). We established a human tendon explant culture system and tested the effects of dexamethasone and triamcinolone on cell viability.

## Materials and methods

### Reagents and culture medium

Dexamethasone sodium phosphate and triamcinolone acetonide were obtained from Sigma (St. Louis, MO). Dulbecco’s modification of Eagle’s Medium (DMEM), penicillin-streptomycin-neomycin, and fetal calf serum (FCS) were supplied by Gibco Laboratories (Grand Island, NY). Alamar blue dye was purchased from Biosource International Inc. (Camarillo, CA). DMEM is a commonly used culture medium derived from Eagle’s Basal Medium, and contains higher concentrations of amino acids and vitamins. FCS provides the proteins essential for cell growth and proliferation. Alamar blue assay is used to monitor changes in cell viability (Voytik-Harbin et al 1998).

Commercially available heat-inactivated FCS was treated with dextran-coated charcoal at 4°C for 12 h before use to minimize the effects of endogenous steroid in the serum. Phenol red-free (PRF) DMEM supplemented with 2% charcoal-stripped FCS was used for all experiments (subsequently referred to as DMEM).

### Human tendon explant culture

The research protocol had been approved by the Human Research Ethics Committee of the authors’ institution. After obtaining informed consent, primary human tendon explant cultures were prepared from healthy hamstring tendon. Strips of tendon (2 mm × 2 mm × 5 mm) were harvested from the hamstring tendon of 12 patients undergoing anterior cruciate ligament reconstruction with hamstring autografts. The number of tendon strips harvested from each patient ranged from 6 to 16. The tissue was rinsed once in phosphate-buffered saline (PBS) containing 1% penicillin-streptomycin-neomycin (PSN), followed by rinsing in PBS. The tendon strips were then transferred to 24-well plates, immersed in 1 mL DMEM, and incubated for 24 h in 5% CO_2_, 95% air at 37°C for stabilization.

At the start of the study, 10 tendon strips obtained from a single patient were used to measure the baseline cell viability and changes after 4 days of culture. The tendon strips showed a large degree of variation in baseline and post-culture Alamar blue fluorescence intensities. The donor identity of each tendon strip was recorded.

### Glucocorticoid treatment

The tendon explants were randomly allocated to different steroid treatments. The tendon explants were transferred to culture plates containing 0, 10, or 100 μM dexamethasone sodium phosphate or containing 0, 10, or 100 μM triamcino-lone acetonide in DMEM, and incubated further for 96 h. The culture medium was changed every 2 days.

### Alamar blue assay

Cell viability in the tendon explants was measured by Alamar blue assay (Springer et al. 1998) before and after glucocorticoid treatment. The tendon explants were incubated with DMEM containing 10% (v/v) Alamar blue for 3 h at 37°C. After 3 h, 200-μL reaction mixtures were pipetted out and transferred to 96-well plates for measurement of fluorescence. The fluorescence intensity of Alamar blue was measured with a fluorometer (Molecular Devices, Sunnyvale, CA) with an excitation wavelength of 530 nm and an emission wavelength of 590 nm. DMEM with 10% Alamar blue, without any tendon explant, was used as reagent blank for the fluorometric measurements. The tendon explants were rinsed thoroughly in PBS to remove any residual Alamar blue dye after each assay.

### Statistics

Fluorescence intensities of Alamar blue were measured in arbitrary fluorescence units. Fluorescence intensities of tendon explants after dexamethasone or triamcinolone treatment were expressed as percentages of activities measured in the same tendon explants before treatment. Statistical analysis was performed using mixed-effects ANOVA, with dosage as a fixed factor and donor identity as a random factor, followed by post hoc Dunnett t-tests. All statistical tests were performed using SPSS software version 15.0. Differences were considered statistically significant at p < 0.05.

## Results

**Figure 1.**
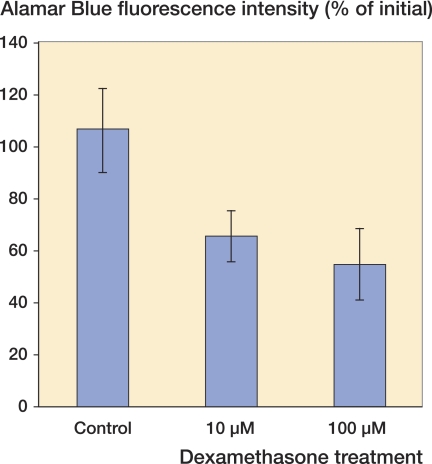
Incubation of human tendon explants with 10 μM and 100 μM dexamethasone for 96 h reduced the fluorescence intensity of Alamar blue to mean 65% (SE 10) and 55% (SE 14) of the original values (n = 15 and n = 16, respectively). Mixed-effects ANOVA and post hoc tests showed significant suppression compared to the controls, the fluorescence intensity of which increased to mean 106% (SE 16) (n = 14).

**Figure 2.**
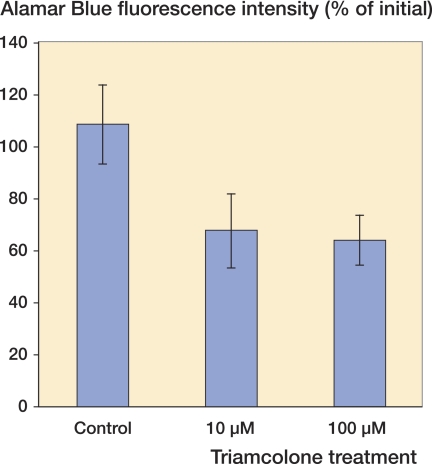
Incubation of human tendon explants with 10 μM and 100 μM triamcinolone for 96 h reduced the fluorescence intensity of Alamar blue to mean 67% (SE 15) and 64% (SE 9) of the original values (n = 19 and n= 21, respectively). Mixed-effects ANOVA and post hoc tests showed significant suppression compared to the controls, the fluorescence intensity of which increased to mean 109% (SE 15) (n = 17).

After 96 h, the fluorescence intensity of Alamar blue in control cultures increased by 6% (n = 14), whereas incubation with 10 μM or 100 μM dexamethasone reduced the fluorescence intensities by 35% (n = 15) and 45% (n = 16), respectively (p = 0.01, mixed-effects ANOVA) (Figure 1). Similar changes were observed with triamcinolone. Incubation of human tendon explants with 10 μM or 100 μM triamcinolone for 96 h reduced the fluorescence intensity of Alamar blue by 33% (n = 19) and 36% (n = 21), respectively, which can be compared to a 9% increase in control cultures (n = 17) (p = 0.07, mixed-effects ANOVA) (Figure 2). Post hoc Dunnett t-tests showed suppression of Alamar blue fluorescence intensity at 10 μM (p = 0.006) and 100 μM (p = 0.004) dexamethasone relative to the controls, and suppression at 10 μM (p = 0.05) and 100 μM (p = 0.04) triamcinolone relative to the controls. There was no significant difference in effect between 10 and 100 μM dexamethasone, and between 10 and 100 μM triamcinolone.

## Discussion

Cell cultures are commonly used to test the response of cells to drugs or other stimuli. The absence of extracellular matrix and of three-dimensional structure in tendon cell cultures may affect the response of tenocytes (Clark et al. 1997, Fu et al. 2005). Assessment of matrix integrity is also impossible in cell cultures. Tendon explant cultures, on the other hand, retain the three-dimensional structure and architectural characteristics of the tissue in vivo. The presence of extracellular matrix is preserved, both in terms of its normal quantity and distribution. Tendon explant cultures reflect the in vivo situation, yet allow one to perform in vitro experiments under controlled conditions. It therefore has a significant advantage over cell cultures—by reflecting the actual changes taking place in vivo.

Tendon explant cultures have been used to study the metabolism of tendon tissue matrix (Abrahamsson et al. 1991, Koob and Vogel 1987). Previous studies using rabbit and bovine tendon explant cultures have shown that normal histomorpho-logical features are maintained in the explants for more than 3 weeks (Koob and Vogel 1987, Abrahamsson et al. 1991). We have been able to maintain human tendon explants in culture for over 10 days, with preservation of typical normal tendon histomorphology. Tenocytes in cultured human tendon explants continued to survive and proliferate, as shown by positive staining of proliferative cell nuclear antigen. Culture for longer periods may also be possible, but this was not attempted in the present study.

Our findings indicate that Alamar blue can be used to detect changes in cell viability in response to glucocorticoid treatment in human tendon explant cultures. Cell viability assays detect cellular metabolic functions by measuring the activity of esterases or oxidative enzymes, e.g. MTT (3-(4,5-dimethylthiazol-2-yl)-2,5-diphenyltetrazolium bromide, a tetrazole) assay measures the reduction of this yellow compound to purple formazan by mitochondrial enzymes. The MTT colori-metric assay can be used to measure changes in cellular activity and cell viability. Alamar blue is another oxidation-reduction sensitive dye that can be used to measure mitochondrial metabolic activity. The oxidized form of Alamar blue, which is dark blue, becomes reduced and turns red when taken into cells. The reduced form of Alamar blue is highly fluorescent. The extent of reduction is a reflection of cellular activity, and can be measured by either colorimetric or fluorometric methods. The assay provides a quick and quantitative index of cellular viability, cell growth, and survival (Voytik-Harbin et al. 1998). It has been used to study cell proliferation in a tumor cell line and also the effects of chemotherapeutic drugs (Pagé et al. 1993).

The use of Alamar blue has several advantages over other existing methods. The procedure is simple and can be performed quickly. It does not interfere with cell metabolism, and there is no involvement of radioactive substances. As Alamar blue dye is not toxic, and the assay is not destructive to cell and tissue samples, repeated measurements of the same sample over time can be performed. It has been shown to be a sensitive method for measurement of metabolic activity in isolated tissue (Springer et al. 1998). All these properties made it an ideal choice for our current study of the effects of glucocorticoids on the cell viability of human tendon explants.

We found that dexamethasone and triamcinolone do have a suppressive effect on cell viability in human tendon within its natural 3-dimensional environment, with matrix anchorage. The suppressive effects of dexamethasone and triamcinolone on cell viability found here are consistent with our previous findings using human tenocyte cell cultures (Wong et al. 2003, 2004). Tenocytes are the major cell type found in tendon; they are responsible for the synthesis and maintenance of collagen and other components of the extracellular matrix, and thus the structural integrity of tendon. Proliferation of tenocytes is an important initial step in tendon healing (Chan et al. 1997). Tenocyte proliferation is followed by deposition of extracelluar matrix, matrix remodeling, and collagen crosslinks (Chan et al. 1998). Suppression of tenocyte activity may affect the normal healing response, resulting in altered matrix synthesis and modulation. In particular, the disturbance of proteoglycan production is of particular significance, as proteoglycans play an important role in the regulation of collagen fibrillogenesis (Scott et al. 1981, Scott and Hughes 1986) and contribute to the viscoelastic properties of tendon (Elliott et al. 2003). The suppression of cell viability in human tendon by dexamethasone and triamcinolone may lead to altered integrity and strength of tendon, predisposing it to spontaneous rupture.

Mixed-effects ANOVA was used in the data analysis to take into consideration the effect of donors who gave multiple tendon strips. The effect of dexamethasone treatment remained statistically significant, irrespective of donor identity. The p-value for triamcinolone treatment was 0.07, which is close to but does not reach the usual cutoff for statistical significance. One possible explanation was that the observed power of analysis for triamcinolone dose was 0.53, which was on the low side with the possible existence of type-II error. A second explanation may be that triamcinolone has less than one-fifth of the glucocorticoid effect of dexamethasone (British National Formulary 2003). The 10 and 100 μM doses of triamcinolone—although corresponding to those of dexamethisone—might result in less suppression, thus making the statistical significance borderline.

The range of glucocorticoid concentration near to the reported serum concentration after oral ingestion of dexamethasone (Loew et al. 1986) was tested in this study. Medical preparations of dexamethasone and triamcinolone acetonide for local injection have concentrations over 10 mM. The tissue levels immediately after local glucocorticoid injection will be near to the concentrations in the preparation, which are much higher than the 10 μM and 100 μM tested in this study. The subsequent change in tissue concentration is difficult to estimate.

Despite the common concern about the risk of spontaneous tendon rupture after local glucocorticoid injection, previous studies in animals have failed to show a definitive relationship and the role of glucocorticoid in tendon rupture remains controversial. The establishment of a human tendon explant culture system and demonstration that it can be used to test the effects of glucocorticoids provides a model for further study. Future investigations on changes in extracellular matrix synthesis, matrix degradation, maturation, and mechanical strength of human tendon after glucocorticoids may provide further information on the actual effect of glucocorticoids on the structural integrity and mechanical properties of tendon.

Human tendon explants provide a good system for the in vitro study of tissue responses in a 3-dimensional tendon structure with preserved matrix. On the other hand, the system has the usual limitations of in vitro systems, and lacks the whole-body responses found in in vivo studies. The tendon explants used for this study were harvested from normal tendons without either inflammatory or degenerative tendinopathy, where glucocorticoid injections are usually given. As the responses of normal tendon explants may differ from those of pathological tendons, the results obtained here from normal tendon explants must still be interpreted with caution and remain to be put into the perspective of rheumatoid arthritis and soft tissue inflammatory conditions.
